# A Draft Map of *E. coli* Proteoforms

**DOI:** 10.1021/acs.analchem.6c03571

**Published:** 2026-08-25

**Authors:** Jorge A. Colón-Rosado, Fei Fang, Liangliang Sun

**Affiliations:** Department of Chemistry, 3078 Michigan State University, 578 S Shaw Lane, East Lansing, Michigan 48824, United States

## Abstract

Top-down proteomics (TDP) enables direct characterization of intact proteoforms, providing protein-level insights into molecular diversity arising from post-translational modifications and sequence variations. Despite this advantage, proteome coverage in TDP remains limited relative to bottom-up proteomics (BUP). To expand coverage, we developed an integrated multidimensional approach combining sequential protein extraction, size-exclusion chromatography (SEC) fractionation, and capillary zone electrophoresis (CZE)-tandem mass spectrometry (MS/MS) and reversed-phase liquid chromatography (RPLC)-MS/MS. This approach identified 743 proteoform families and 10,613 proteoforms from *E. coli* cells through hundreds of MS runs. By incorporating previous *E. coli* TDP data sets from our group, we identified 14,932 proteoforms from 985 proteoform families, covering 43% of the *E. coli* proteome. The data represent the highest proteome coverage of cells by MS-based TDP, creating a draft map of *E. coli* proteoforms. The results offer strong evidence that MS-based TDP can reach high proteome coverage.

## Graphical abstract

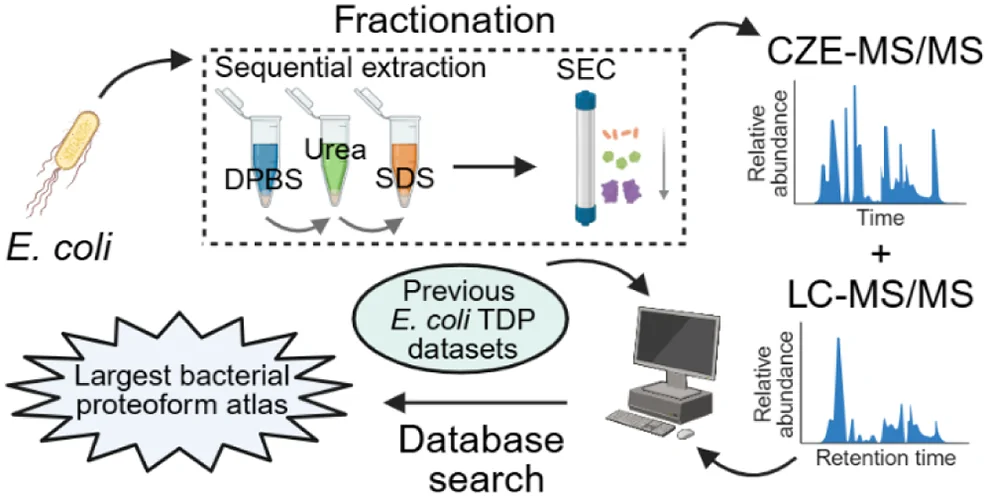

## Introduction

In top-down proteomics (TDP), proteins are extracted from cells or tissues and analyzed via mass spectrometry (MS) and tandem MS (MS/MS).^1,2^ This intact protein analysis enables the characterization of proteoforms, the different molecular forms of a protein that are produced from a single gene. These proteoforms are produced by alternative splicing, genetic variation, and post-translational modifications (PTMs).^3^ Since different proteoforms can have different biological functions,^4^ analyzing the proteoform landscape of an organism enables the identification of specific proteoforms involved in different biological processes such as development, aging, and disease.^5–7^ For example, histone proteoforms with distinct acetylation or methylation states regulate chromatin accessibility and gene expression, whereas tau proteoforms differing in phosphorylation and truncation patterns are associated with neurodegenerative disorders such as Alzheimer’s disease.^8–10^

In contrast, bottom-up proteomics (BUP) involves enzymatic digestion of proteins into peptides prior MS analysis.^11^ This approach offers high throughput and robustness, and its peptide-level nature facilitates separation and ionization, enabling deep proteome coverage.^12,13^ However, after proteins are enzymatically cleaved and peptides generated, information about their precursor proteoforms and their specific PTM combinations and sequence variants is lost.^11^

Although the proteoform-level information captured in TDP provides insights into their molecular diversity and functional specificity, a major limitation of this approach is its relatively low proteome coverage compared to BUP.^11^ While TDP has intact proteoform resolution, comprehensive profiling remains challenging. Analysis of intact proteins is hindered by their intrinsic physicochemical complexity, which complicates solubilization, separation, and ionization. For instance, humans possess ∼20,000 protein-coding genes (PCGs), yet more than one million proteoforms are estimated to be produced.^4,14^ Even in relatively less complex organisms such as *Escherichia coli* (*E. coli*), which contains ∼4,200 PCGs, the number of proteoforms is still estimated to be in the tens of thousands.^15,16^ Moreover, the dynamic range of protein abundance in biological samples can be extremely large, spanning up to 12 orders of magnitude in human plasma and 6 in *E. coli*.^17,18^ Altogether, the high sample complexity of biological systems remains a primary factor limiting proteome coverage in TDP.

To overcome the challenge of limited proteome coverage in TDP, various fractionation methods have been employed to reduce sample complexity before MS analysis. Many of these approaches exploit the physicochemical properties of proteoforms, such as protein solubility.^19–21^ Because proteins differ widely as a result of their structural and chemical features, the composition of the lysis buffer determines which subsets are extracted. Physiological salt buffers such phosphate-buffered saline (PBS) and its variant Dulbecco’s PBS (DPBS) are mild and nondenaturing, preserving native protein structures and primarily extracting soluble cytosolic proteins.^22^ In contrast, chaotropic buffers such as urea are typically used at high concentrations (∼8 M) to disrupt hydrogen bonding and weaken hydrophobic interactions, thereby unfolding proteins and enhancing solubilization of complex or aggregated proteins.^23–25^ Finally, sodium dodecyl sulfate (SDS) is a strong anionic detergent that efficiently disrupts lipid bilayers, enabling the recovery of membrane-associated and highly hydrophobic proteins.^26,27^

Although lysis buffers fractionate proteins according to their solubility, additional fractionation steps are often necessary to further reduce sample complexity. Among these, size-exclusion chromatography (SEC) separates proteoforms according to their hydrodynamic radius as they travel through a porous stationary phase.^28^ During separation, larger proteoforms elute first because they are excluded from the pores while smaller proteoforms penetrate the pores and elute later. Due to its gentle and reproducible nature, SEC has been extensively used in TDP workflows to reduce sample complexity prior to MS analysis.^29,30^

After fractionation, proteoforms in each fraction are separated by a liquid-phase method and detected via tandem mass spectrometry (MS/MS).^11^ Reversed-phase liquid chromatography (RPLC)-MS/MS remains the most widely used approach in TDP due to its reproducibility and robustness.^31^ In contrast, capillary zone electrophoresis (CZE)-MS/MS, has demonstrated superior sensitivity than RPLC-MS/MS, particularly for mass-limited samples, owing to its low sample loss and exceptionally high separation efficiency. For example, CZE-MS/MS achieved a comparable number of calf histone proteoforms with RPLC-MS/MS (152 vs 176), even though the protein amount injected in CZE was 30-fold less than in RPLC (75 ng vs 3 μg).^9^ In addition, CZE-MS/MS and RPLC-MS/MS are complementary in proteoform identifications due to their orthogonal separation mechanisms, making their combination powerful for comprehensive TDP analyses.^32,33^

The combination of multidimensional fractionation and orthogonal separations substantially enhances proteome coverage in TDP by reducing sample complexity.^34^ For example, combining SEC and RPLC prefractionation followed by CZE-MS/MS enabled identification of 5,705 proteoforms in *E. coli*, representing the largest bacterial TDP data set.^15^ The combination of high-field asymmetric ion mobility spectrometry (FAIMS) and CZE-MS/MS improved the proteome coverage of TDP substantially.^35,36^ Serial SEC followed by RPLC-MS/MS achieved a 15-fold increase in the detection of higher mass proteins (up to 223 kDa) compared to RPLC alone.^28^

Building upon these advances, we aim to develop an integrated approach to boost proteome coverage of TDP. Our approach couples sequential protein extraction using buffers of increasing denaturing strength (DPBS, urea, and SDS) with SEC fractionation, followed by CZE-MS/MS and RPLC-MS/MS for proteoform characterization of *E. coli* cells. *E. coli* was selected as a model organism in this study due to its well-annotated genome, small proteome size (around 2,000 expressed proteins in a specific condition), and rapid and cost-effective growth.^27^ In addition, previously published and unpublished TDP data sets from our group were incorporated to further expand proteome coverage.^15,34^ Through this multidimensional strategy, our goal is to enhance proteome coverage in TDP and establish the most comprehensive bacterial proteoform atlas to date using *E. coli*.

## Experimental Section

### Materials and Reagents

Hydrofluoric acid (HF, 48–51% solution in H_2_O) and acrylamide were purchased from Acros Organics (NJ, USA). LC/MS-grade ammonium hydroxide (NH_4_OH) was purchased from Fisher Chemical (Thermo Fisher Scientific, NJ, USA). Sodium hydroxide (pellets) was obtained from Macron Fine Chemicals (PA, USA). Sodium dodecyl sulfate (SDS), Dulbecco’s phosphate-buffered saline (DPBS), dithiothreitol (DTT), and iodoacetamide (IAA) were purchased from Sigma-Aldrich (MO, USA). Amicon Ultra centrifugal filters (0.5 mL, 10 kDa molecular weight cutoff, MWCO) were purchased from Millipore Sigma (MA, USA). PhosSTOP and cOmplete Tablets EASYpack protease inhibitors were obtained from Roche. Bare fused-silica capillaries (50 μm i.d. × 360 μm o.d.) were purchased from Polymicro Technologies (AZ, USA). Hydrochloric acid (HCl) and the BCA Protein Assay Kit–Reducing Agent Compatible were obtained from Thermo Fisher Scientific (MA, USA). Ammonium acetate (NH_4_CH_3_CO_2_) was purchased from Invitrogen (Thermo Fisher Scientific, USA). LC/MS-grade water, acetonitrile (ACN), methanol (MeOH), and formic acid (FA) were obtained from Fisher Scientific (PA, USA). Nalgene Rapid-Flow filter units (0.2 μm CN membrane, 50 mm diameter) used for aqueous mixture filtration, as well as urea and HPLC-grade chloroform, were purchased from Thermo Scientific (MA, USA).

### Cell Culture

*Escherichia coli* (*E. coli*, K-12 strain, MG1655 substrain) was cultured in Terrific Broth (TB) media at 37 °C with shaking at 177 rpm. Cells were harvested at an OD_600_ value of ∼0.7 by centrifugation with an Allegra X-30R centrifuge (Beckman Coulter, CA, USA) at 3,000 × g for 15 min at 4 °C. The resulting cell pellet was washed three times with DPBS and stored at −80 °C until use.

### Sequential Protein Extraction and Sample Preparation

To maximize proteome coverage, *E. coli* proteins were sequentially extracted using DPBS, 8 M urea, and 2% (w/v) SDS. 1.11 g of *E. coli* cell pellet was processed using the integrated multidimensional workflow. For each extraction step, 5.55 mL of lysis buffer containing protease and phosphatase inhibitors (25 mg each) was used. The cell pellet was first resuspended in DPBS, homogenized on ice for a total of 3 min with a Fisherbrand 150 Homogenizer (Fisher Scientific, PA, USA), and lysed on ice for 12 min by sonication with a Branson Sonifier 250 from (VWR Scientific, IL, USA). The lysate was then centrifuged at 16,000 × g for 30 min at 14 °C, and the supernatant containing the extracted proteins was collected. The remaining pellet was re-extracted sequentially with urea and SDS buffers following the same procedure (Figure 1).

**Figure 1:**
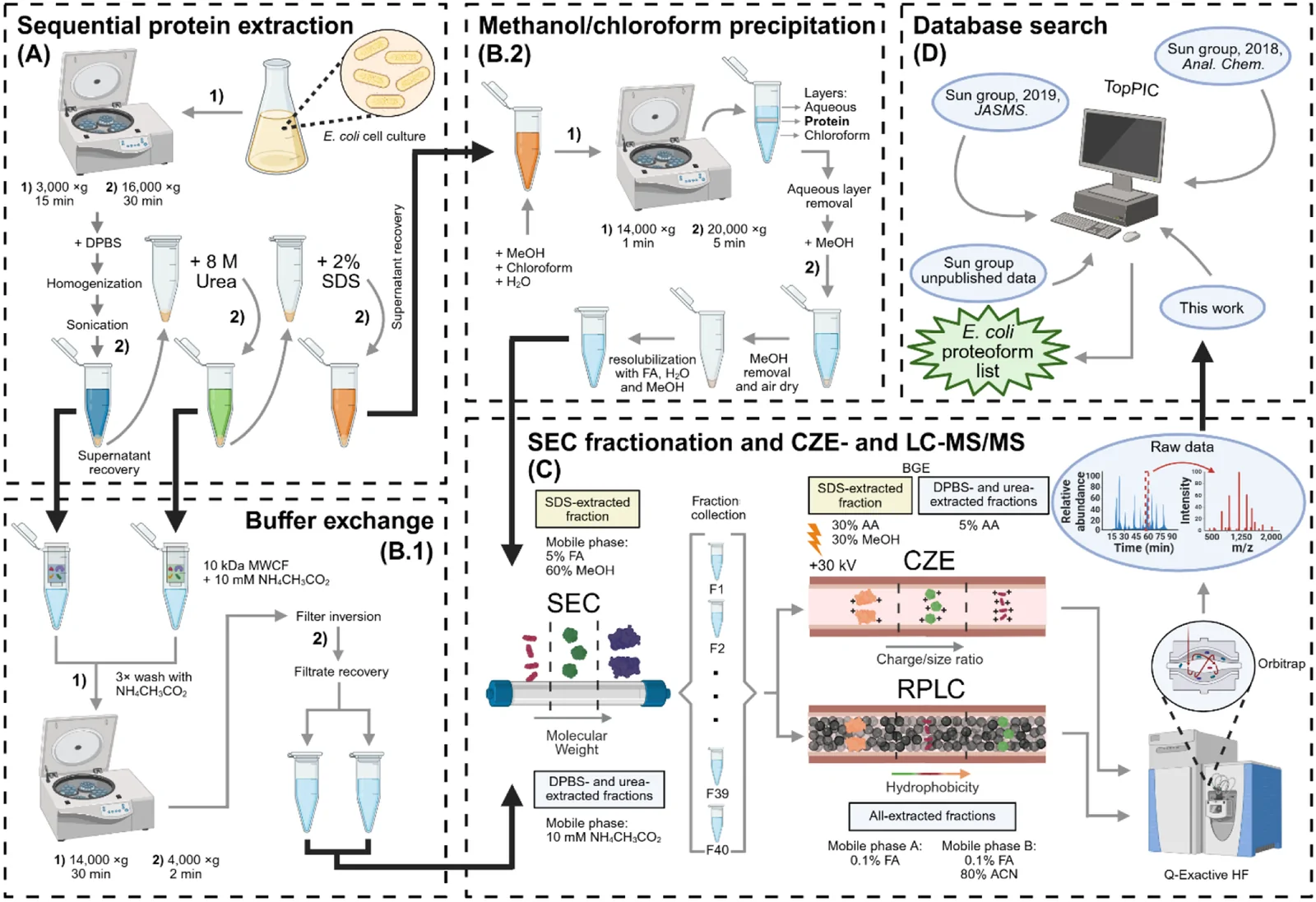
Overview of the sequential protein extraction workflow. A) *E. coli* cells were sequentially extracted with three lysis buffers (DPBS, urea, and SDS) using the same cell pellet. B.1) DPBS- and urea-extracted lysates were cleaned by buffer exchange using 10 kDa MWCO filters and 10 mM NH_4_CH_3_CO_2_. B.2) SDS-extracted lysates were purified by methanol/chloroform precipitation, followed by resolubilization in FA/MeOH/H_2_O. C) Processed extracts were fractionated by SEC and subsequently analyzed by CZE- and RPLC-MS/MS for proteoform characterization. D) Data from this study were combined with previously published and unpublished*E. coli*TDP data sets from the Sun group to generate the draft map of*E. coli*proteoforms.

Two sample cleanup strategies were applied depending on the extraction buffer: buffer exchange using MWCO filters for the DPBS and urea extracts, and methanol/chloroform precipitation for the SDS extract. Buffer exchange effectively removes nonvolatile salts and residual reagents from the DPBS and urea lysates, whereas methanol/chloroform precipitation was selected for SDS extracts because it is more efficient in SDS and lipid removal compared to MWCO-based cleanup.^37,38^ Protein concentrations were determined using the BCA assay.

For methanol/chloroform precipitation, 100 μL of protein solution (∼100 μg) was mixed with 400 μL MeOH and 100 μL chloroform in a 1.5 mL microcentrifuge tube, vortexing after each addition. Subsequently, 300 μL of H_2_O was added, and the mixture was vortexed until protein precipitation became visible as a cloudy interface. Samples were then centrifuged at 14,000 × g for 1 min to form a protein disk at the aqueous and organic interface. The upper aqueous layer was discarded, and the interface was washed with 400 μL MeOH. After centrifugation at 20,000 × g for 5 min, the residual solvent was removed, and the protein pellet was air-dried and stored at −20 °C until downstream analysis. The pellet was resolubilized using a protocol optimized for recovering membrane-associated and highly hydrophobic proteins, which generally require strongly acidic conditions.^21^ Briefly, the protein pellet was dissolved in 20 μL of prechilled 90% FA, immediately diluted into 60 μL 3:6:1 FA/MeOH/H_2_O (v/v), and ultrasonicated on ice for 5 min.

For MWCO buffer exchange, DPBS- and urea-extracted lysates were exchanged into 10 mM NH_4_CH_3_CO_2_ (pH = 7). Lysates were loaded onto 10 kDa filter units and centrifuged at 14,000 × g for ∼30 min at 4 °C for DPBS extracted samples and 14 °C for urea extracted samples until concentrated to <50 μL. Subsequently, 200 μL of 10 mM NH_4_CH_3_CO_2_ was added, mixed by gentle pipetting, and centrifuged under the same conditions. This wash step was repeated three times, and the final retentate (∼30 μL) was collected by inverting the filter unit and centrifuging at 3,000 × g for 3 min. The resulting protein solution was stored at −20 °C until downstream analysis.

Each extraction condition (DPBS, urea, and SDS) was processed in triplicate, and replicate samples were pooled prior to SEC fractionation to reach the 100 μL injection volume per extraction buffer.

### Peptide Sample Preparation

A BUP experiment was performed to obtain a comprehensive representation of the expressed *E. coli* proteome, as this approach provides broader and more representative protein identifications compared to intact protein analysis. A 0.586 g of *E. coli* cell pellet was lysed in 8 M urea and 100 mM NH_4_HCO_3_ (pH = 8) using the same homogenization conditions as in intact protein analysis. After cell lysis, the sample was centrifuged at 14,000 × g for 30 min at 14 °C, and the supernatant containing the proteins was collected. Protein concentrations were determined using the BCA assay.

Samples were denatured at 37 °C for 30 min, reduced with 1 μL of 1 M DTT for 20 min and, alkylated with 4 μL of 1 M IAA for 20 min at room temperature in the dark. Residual IAA was quenched by adding 1 μL of 1 M DTT and stored at room temperature for 10 min. Samples were then diluted 4-fold with 100 mM NH_4_HCO_3_ to reduce urea concentration to ∼2 M, compatible with trypsin activity. Enzymatic digestion was performed by adding 1 μL of trypsin (0.48 μg/μL) and incubating at 37 °C for 12 h. Digestion was quenched by adding 1 μL of 20% (v/v) FA, and samples were stored at −80 °C until use.

### SEC for Intact Protein Fractionation

SEC fractionation was performed using an Agilent 1260 Infinity II HPLC system equipped with a UV–Vis detector set to 254 nm. For the SDS-extracted lysate, the separation was carried out using 5% FA and 60% MeOH (v/v, pH = 2) as the mobile phase, whereas DPBS- and urea-extracted lysates were separated using 10 mM NH_4_CH_3_CO_2_ as the mobile phase. 100 μL of each lysate was injected into an Agilent column (4.6 × 300 mm, 5 μm, 500 Å) at a flow rate of 200 μL/min. Fractions were collected every 30 s over a 30 min separation, beginning at the first UV–vis absorbance peak. Protein samples were concentrated to a final volume of ∼30 μL by speed vacuum with a Savant SPD131DDA SpeedVac Concentrator (Thermo Scientific, MA, USA). Fractions were stored at −80 °C until use.

### High-pH RPLC for Peptide Fractionation

Peptide fractionation was performed under high-pH conditions using an Agilent column (3.0 mm × 15 cm, 2.7 μm, 120 Å; InfinityLab Poroshell 120 EC-C18). Mobile phase A consisted of 5 mM NH_4_HCO_3_, and mobile phase B consisted of 5 mM NH_4_HCO_3_ in 80% ACN, with both adjusted to pH = 9 with NH_4_OH. Peptides were separated using a flow rate of 200 μL/min with the following gradient: 0% B was held for 5 min, increased from 0 to 8% B in 2 min, 8 to 50% B in 30 min, 50 to 80% in 3 min, and maintained at 80% B for 20 min. Fractions were collected every 1 min over a 60 min separation, beginning at the first UV–vis absorbance peak. A total of 46 fractions were collected and subsequently combined, yielding 23 pooled fractions (F1+F24, F2+F25··· F23+F46).

### CZE

CZE was performed using a Beckman Coulter CESI 8000 Plus CE system (SCIEX, MA, USA). The 1-m-long capillary (50 μm i.d. × 360 μm o.d.) used for CZE was coated with linear polyacrylamide (LPA) following previously described protocols.^39,40^ The third-generation electrokinetically pumped sheath flow CE-MS interface (EMASSII CE-MS interface; CMP Scientific, NY, USA) was used to couple CZE to MS.^41,42^ The capillary tip was etched with HF for 90 min to reduce the distance between the capillary outlet and emitter orifice, minimizing sample dilution. For SDS-extracted proteins, the background electrolyte (BGE) consisted of 30% AA and 30% MeOH, while for DPBS- and urea-extracted proteins, 5% AA (pH = 2.3) was used. The sheath buffer contained 10% MeOH and 0.2% FA.

A voltage of 30 kV was applied at the injection end for separation. For SDS-extracted samples only, supplemental pressure was applied as follows: 0.1 psi (0–50 min), 0.5 psi (50–60 min), and 10 psi (60–70 min) for flushing. The electrospray voltage was maintained at 2.2–2.4 kV on the sheath buffer for electrospray ionization. 200 nL of the samples were loaded into the capillary. Electrospray emitters with a 20–30 μm opening were fabricated using a P-1000 Micropipette Puller (Sutter Instrument, CA, USA).

### Low-pH RPLC

An EASY nanoLC-1200 (Thermo Scientific, MA, USA) was used for proteoform and peptide separation prior to MS/MS analysis. For intact protein separation, an in-house packed analytical column (75 μm i.d. × 20 cm, C4, 3 μm, 300 Å; Sepax Technologies, DE, USA) was used. One μL of sample was loaded onto the column and separated at a flow rate of 400 nL/min using the following gradient: increased from 2 to 15% B in 5 min, 15 to 80% B in 53 min, maintained at 80% B for 20 min, decreased from 80 to 2% in 2 min, and maintained at 2% for 10 min.

For peptide separation, another in-house packed analytical column (75 μm i.d. × 20 cm, C18, 1.9 μm, 120 Å; ESI Source Solutions, MA, USA) was employed. Buffer A consisted of 0.1% (v/v) FA, and buffer B consisted of 80% ACN and 0.1% FA. 0.5 μL injection of sample was loaded and separated at a flow rate of 200 nL/min and the following gradient: increased from 2 to 8% B in 5 min, 8 to 40% B in 65 min, 40% to 80% in 10 min, and maintained at 80% for 10 min.

### MS

A Q-Exactive HF (Thermo Fisher Scientific, NJ, USA) mass spectrometer was used for MS and MS/MS analysis. For TDP, the full MS (MS1) scans were acquired from 500–2,000 *m*/*z* at a resolution of 120,000 (at *m*/*z* 400) with an AGC target of 3E6 and maximum injection time of 50 ms. MS/MS (MS2) spectra were acquired at a resolution of 60,000 with an AGC target of 1E5 and a maximum injection time of 100 ms. The loop count was set to 5 (top 5). The quadrupole isolation window was 2.0 *m*/*z*, and the normalized collision energy (NCE) was set to 20. The ion intensity threshold was set to 2.0E4, and the dynamic exclusion window was 30 s.

For BUP, the MS1 scan range was 350–1,800 *m*/*z* at a resolution of 60,000 with an AGC target of 3E6. MS2 spectra were also acquired at a resolution of 60,000 with an AGC target of 1E5. The maximum injection times were 50 ms for MS1 and 150 ms for MS2. The loop count was set to 10 (top 10). The quadrupole isolation window was set to 2.0 *m*/*z* and the NCE was set to 28. The ion intensity threshold was 6.7E4 and the dynamic exclusion window was 15 s.

We also generated a data set consisting of low-resolution MS1 and high-resolution MS2 spectra for fractions containing proteins >30 kDa using an Orbitrap Exploris 480 (Thermo Fisher Scientific, NJ, USA) mass spectrometer. MS1 scans were acquired from 500–2,500 *m*/*z* at a resolution of 7,500 (at *m*/*z* 200) with a normalized AGC target of 100% and the maximum injection time was set to auto. MS2 spectra were acquired at a resolution of 120,000 with a normalized AGC target of 100% and the maximum injection time was set to auto. The number of dependent scans was set to 6. The quadrupole isolation window was 2.0 *m*/*z*, and the normalized collision energy (NCE) was set to 25%. The ion intensity threshold was set to 1.0E4, and the dynamic exclusion window was 30 s.

### Database Search

For intact protein analysis, raw data files were converted to mzML format using MSConvert from the ProteoWizard Toolkit.^43^ The resulting mzML files were deconvoluted with TopFD (Top-down mass spectral Feature Detection) and searched using TopPIC (Top-down mass spectrometry based Proteoform Identification and Characterization, v 1.7.8).^44,45^ The *Escherichia coli* (strain-K12) UniProt reference proteome (UP000000625) was used as the search database. Variable modifications included oxidation (M), phosphorylation (S, T, Y), acetylation (protein N-terminus, K), and methylation (K, R). The mass tolerance was set to 10 ppm, and the maximum number of variable modifications was set to 2. The false discovery rate (FDR) was performed using a target-decoy database approach. Spectrum- and proteoform-level identifications were filtered with 1% FDR.

The same parameters were applied when combining TDP files with TopPIC for database searches across extraction buffers (DPBS, urea, and SDS) and separation methods (CZE and LC).

For BUP, peptide database search was performed using MSFragger v 22.0.^46^ Both precursor and fragment ion mass tolerances were set to 20 ppm, and trypsin was specified as the digestion enzyme. Variable modifications included oxidation (M), acetylation (N-terminal, K), and phosphorylation (S, T, Y). Carbamidomethylation (C) was set as a fixed modification. Peptide- and protein-level identifications were filtered with 1% FDR.

### Data Analysis

Gene Ontology (GO) enrichment analysis was performed using the Database for Annotation, Visualization and Integrated Discovery (DAVID) bioinformatics resources.^47^

### Higher Mass Proteoform Analysis

Mass deconvolution of higher mass proteoforms (>30 kDa) was performed with UniDec, a universal Bayesian zero-charge deconvolution software.^48^ MS1 spectra of proteoforms with well-defined charge state envelopes were manually selected, and the mass and charge ranges were adjusted individually for each proteoform during deconvolution. To demonstrate the feasibility of identifying higher mass proteoforms, two representative proteoforms were further analyzed using ProSight Native through the Top-down Search module from the Top-down Characterization suite.^49^ For deconvolution, the maximum number of masses per spectrum was set to 1, and both mass and charge ranges were adjusted according to each proteoform’s MS1 profile. The deconvolution algorithm was set to auto with the latest kDecon version enabled. Fragmentation maps were visualized and annotated using ProSight Lite.^50^

### Proteoform Confidence Classification

Proteoforms were classified using a custom Python script following the five-level proteoform classification proposed by the Consortium for Top-Down Proteomics.^51^ The script parsed the TopPIC proteoform output and evaluated ambiguity based on the information available in the summarized results. PTM localization ambiguity was assigned when multiple possible modification sites were reported in the variable PTMs or unexpected modifications fields. PTM identity ambiguity was assigned when an unexplained mass shift was reported in the unexpected modifications field. Potential gene of origin ambiguity was assigned when TopPIC reported more than one protein hit for a proteoform. Amino acid sequence ambiguity could not be directly assessed from the summarized TopPIC output and was therefore not included in the classification. Proteoforms without detected ambiguity were classified as Level 1. Proteoforms containing a single ambiguity were classified as Level 2A (PTM localization), Level 2B (PTM identity), or Level 2D (potential gene of origin). Proteoforms containing two or three ambiguity categories were classified as Levels 3 and 4, respectively.

## Results and Discussion

After applying our sequential extraction and SEC fractionation workflow, we evaluated proteome coverage obtained from each separation platform (CZE-MS and RPLC-MS). With CZE-MS, urea lysate produced the largest number of identifications (530 proteoform families; 5,299 proteoforms), consistent with its superior denaturing and solubilizing properties for cytosolic proteins, which accounts for ∼60% of the *E. coli* proteome, Figure 2.^52,53^ DPBS yielded intermediate coverage (330 proteoform families; 2,950 proteoforms), whereas SDS produced the fewest identifications (97 proteoform families; 1,299 proteoforms). This result reflects the sequential nature of the extraction workflow, in which some hydrophobic proteoforms may have been recovered during the preceding DPBS and urea extraction steps, as well as incomplete resolubilization following detergent removal prior to MS analysis.

**Figure 2:**
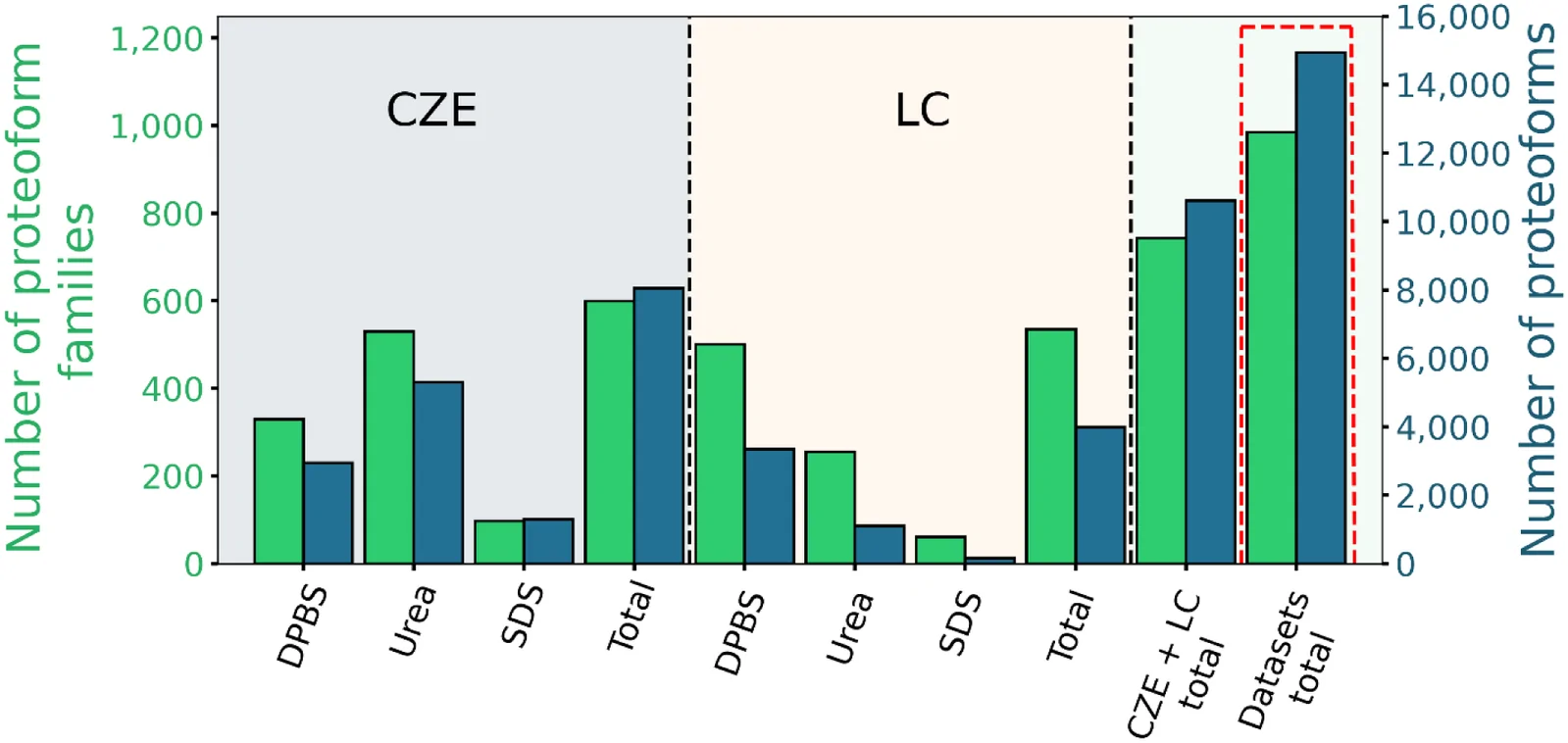
Proteoform families and proteoform identifications from*E. coli* using the sequential protein extraction workflow. The number of identified proteoform families (green) and proteoforms (blue) are shown for each extraction buffer (DPBS, urea, and SDS) and separation method (CZE and LC). Bars labeled “Total” represent the unique identifications obtained from each separation technique, while “CZE + LC total” indicates the combined nonredundant data set. Integration of previous *E. coli* TDP data sets^15,34,54^ further increased the total number of identifications, highlighted by the red dashed box.

After performing a combined search across all three lysis buffers, CZE-MS produced a total of 599 proteoform families and 8,051 proteoforms, representing more than 40% increase in the number of proteoform identifications compared to the urea method alone (Supporting Information II). The greater number of identifications from urea also underscores its higher protein extraction efficiency compared to DPBS, despite sequential extraction starting with the latter. When RPLC-MS was used, the distribution of identifications shifted relative to CZE-MS. DPBS extraction yielded the highest number of identifications (501 proteoform families; 3,345 proteoforms), followed by urea (255 proteoform families; 1,106 proteoforms) and SDS (61 proteoform families; 161 proteoforms), Figure 2. Combining the identifications from all three extraction buffers yielded 535 proteoform families and 3,989 unique proteoforms, representing about a 20% improvement in proteoform identifications compared to DPBS alone (Supporting Information II). The results indicate that combining various proteoform extraction approaches improves proteoform coverage.

CZE identified more proteoform families and proteoforms than RPLC, with 377 proteoform families and ∼1400 proteoforms shared between methods. Combining RPLC and CZE data, a total of 743 unique proteoform families and 10,613 unique proteoforms were identified, Figure 2. The number of proteoform families and proteoform identifications from the combined approach is improved by >35% and >160%, respectively, compared to those from RPLC-MS alone. The results highlight the complementarity of CZE-MS and RPLC-MS in TDP of complex proteomes. We further examined key physicochemical properties of the identified proteoforms from different extraction buffers (DPBS, urea, and SDS) and separation techniques (CZE and LC), including their isoelectric points (pIs), molecular mass, and hydrophobicity (GRAVY), Figure S1. CZE-MS only identified low-mass proteoforms (<15 kDa) whereas LC-MS identified proteoforms close to 30 kDa. No clear differences in pI and GRAVY were observed between CZE and LC. The SDS-extract had proteoforms with higher median mass and pI compared to DPBS and urea extracts according to LC and CZE data. No obvious difference in proteoform hydrophobicity (GRAVY) was observed across the three different extraction buffers. We further studied the number of proteoform and proteoform families’ identifications as a function of the SEC fraction number for different extraction buffers from LC-MS and CZE-MS, Figure S2. Interestingly, LC-MS and CZE-MS produced similar proteoform and proteoform families’ distributions for DPBS and SDS extracts, but substantially different ones for the urea extract. For the urea extract, CZE-MS (Figures S2A and S2B) produced more even distributions of proteoforms and proteoform families across SEC fractions compared to LC-MS (Figures S2C and S2D). The data here provides a better understanding of the complementarity of different extraction buffers and of LC-MS and CZE-MS for proteoform identification.

It is challenging to identify proteoforms larger than 30 kDa using TDP because of their much wider charge-state distributions compared to small proteoforms, resulting in substantially lower signal-to-noise ratios.^55^ As shown in Figure S1, all the proteoforms identified are smaller than 30 kDa. To improve the identification of large proteoforms, we performed separate experiments employing low mass resolution for proteoform measurement and high mass resolution for measurement of fragment ions. Manual inspection of MS1 spectra revealed clear charge-state envelopes for many high mass proteoforms. Figure 3 shows representative MS1 spectra of six large proteoforms in the urea extract. After mass deconvolution with UniDec, proteoforms with masses ranging from 31 kDa up to 71.4 kDa were observed, demonstrating that our approach can effectively measure large proteoforms. To explore this further, we used ProSight Native to identify high-mass proteoforms. Mass deconvolution with the kDecon algorithm was performed on proteoforms from the urea and DPBS extracts. Combined with the parent and fragment ions matched from MS and MS/MS scans, two proteoforms with masses 40,986.96 Da and 71,422.53 Da were identified as phosphoglycerate kinase (PGK; P0A799) and chaperone protein HtpG (P0A6Z3), Figure S3. PGK, a highly abundant glycolytic enzyme in *E. coli*, ranks among the top ∼5% of expressed proteins with an estimated abundance of ∼5,600 ppm according to PaxDb (strain K-12).^56^ In contrast, HtpG is a stress-induced molecular chaperone of the Hsp90 family that ranks among the top ∼10% of expressed *E. coli* proteins (∼766 ppm, PaxDb), reflecting moderate abundance under physiological conditions.^56^ The successful identification of these two large proteoforms, particularly HtpG, highlights the capability of our workflow to characterize proteins well above 30 kDa.

**Figure 3:**
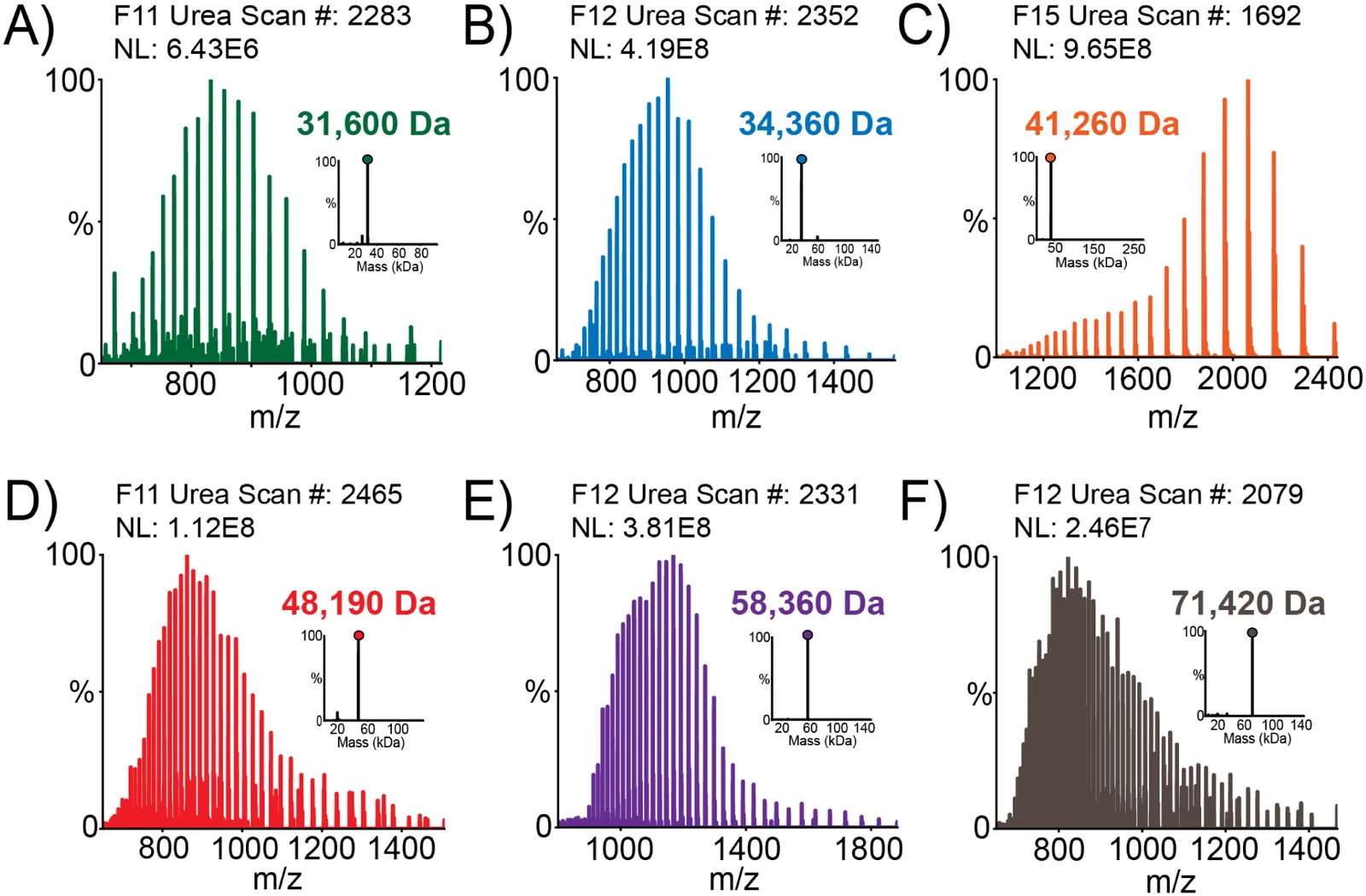
MS1 spectra of high mass (>30 kDa) *E. coli* proteoforms and their corresponding deconvoluted masses. A)–F) show representative MS1 spectra of urea-extracted proteoforms, with deconvoluted masses obtained using UniDec. The detected proteoforms range from 31.6 to 71.4 kDa.

We performed a comprehensive BUP analysis of the*E. coli* cell lysate by 2D-RPLC-MS/MS with the identification of 2,276 protein groups, providing a robust reference for estimating the total expressed proteome of the *E. coli* cells analyzed in this study (Supporting Information II). The 8 M urea extract was selected for BUP because urea is a widely used chaotropic reagent that efficiently solubilizes proteins with diverse physicochemical properties, providing broad proteome coverage suitable for comparison with the combined TDP data set. The identification of 10,613 proteoforms of 743 proteoform families from *E. coli* cells in this study represents about 33% of the *E. coli* proteome by number of identified proteoform families (i.e., 743/2276). After incorporating previous TDP*E. coli* data sets^15,34,54^ to our *E. coli* data, identifications increased to 985 proteoform families and 14,932 proteoforms, representing 33% and 41% increases in proteoform families and proteoforms, respectively, compared to the data set generated in this study. With nearly 1,000 proteoform families and 15,000 proteoforms identified, this combined data set covers approximately 43% of the*E. coli*proteome (i.e., 985/2276) (Supporting Information II). To the best of our knowledge, the data represents the largest bacterial proteoform database and the highest proteome coverage reported to date for TDP of any organism.

We further compared the Gene Ontology (GO) enrichment data between the BUP and TDP data sets (Figure S4). For the biological process and cellular component, BUP and TDP agreed in multiple categories, with a couple of substantial differences. For example, TDP exhibited stronger enrichment for the translation biological process (Figure S4A), consistent with the preferential detection by TDP of abundant and structurally conserved proteoforms involved in translation.^57^ In contrast, BUP demonstrated broader enrichment of cytosolic and cytoplasmic proteoforms in the cellular component category compared to the TDP data set (Figure S4B). Molecular function analysis showed that protein binding-related functions were the most significantly enriched categories in both BUP and TDP data sets, with substantially higher enrichment observed in BUP (Figure S4C). The observed agreement and differences between TDP and BUP are most likely due to the high proteome coverage produced by TDP and the still-limited coverage of large proteoforms (>30 kDa) in the TDP data sets.

Proteoforms identified in this study were classified according to the five-level proteoform classification framework.^51^ Overall, 43.2% of proteoforms were classified as Level 1, indicating no detected ambiguity, whereas Level 3 proteoforms were the most abundant (48.4%). Level 2 proteoforms accounted for 8.3%, while Levels 4 and 5 together represented less than 1% of all identifications (Supporting Information II). Most Level 1 proteoforms were unmodified, with oxidation and methylation being the most frequently observed PTMs, Figure 4.

**Figure 4:**
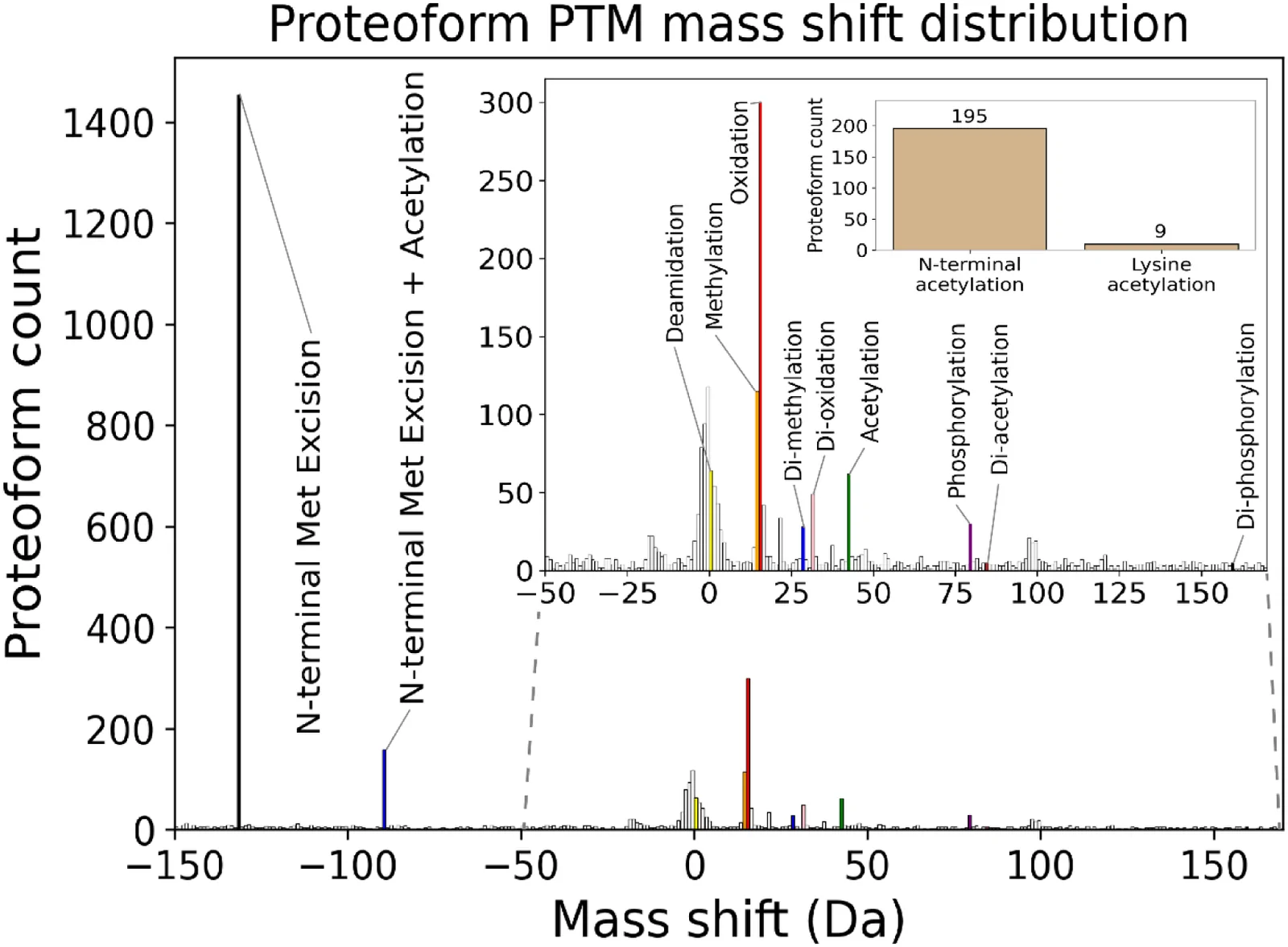
Mass shift distribution of identified proteoforms showing the most abundant PTM mass shifts identified by TDP.

The most abundant modifications identified in this study were N-terminal methionine excision, followed by oxidation and N-terminal methionine excision with acetylation, Figure 4. Additional PTMs included deamidation, acetylation, and phosphorylation. Proteoforms containing PTM combinations, including dimethylation, dioxidation, and diphosphorylation, were also identified. For acetylated proteoforms, approximately 96% corresponded to N-terminal acetylation.

Over the past two decades, continuous methodological advances in TDP have substantially increased proteome coverage across different organisms, Figure 5. Early pioneering studies in the late 1990s and early 2000s by the McLafferty and Kelleher groups marked the advent of TDP for bacterial and yeast proteomes.^58,59^ Subsequent developments in sample preparation, multidimensional fractionation, and high-resolution MS have led to remarkable improvements, exemplified by the works of the Sun, Wang, and Tholey groups.^15,22,60,61^

**Figure 5:**
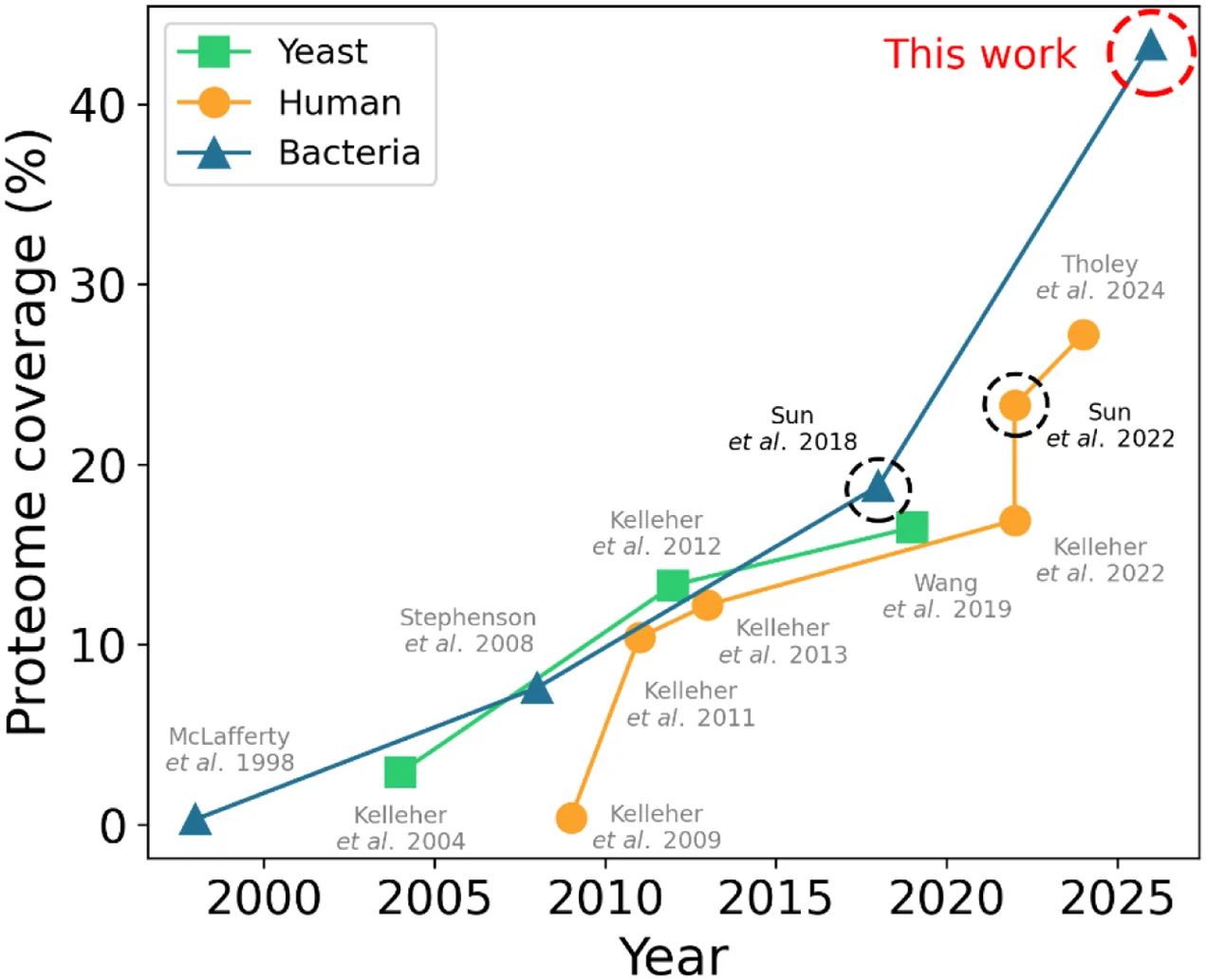
Bacterial, yeast, and human proteome coverage trends by TDP over the last nearly 30 years. The data sets used to produce this figure are from references 15,22,58–61 , and^65–70^ .

In the present study, our integrated multidimensional workflow achieved approximately 43% proteome coverage in*E. coli* when complemented with previous TDP data sets, representing the most comprehensive bacterial proteome characterization to date by the TDP approach. We expect that the continual innovation in sample preparation, separation, and MS technologies will further advance the depth of proteoform and proteome coverage across various biological systems.

## Conclusions

This multidimensional TDP workflow, combined with previous*E. coli* TDP data sets, enabled the identification of 985 proteoform families and 14,932 proteoforms, representing the most comprehensive proteoform atlas reported for*E. coli* to date. By integrating sequential extraction, SEC fractionation, and orthogonal CZE- and LC-MS/MS, this study demonstrates the effectiveness of multidimensional strategies in expanding proteoform coverage in *E. coli*.

Despite this achievement, the workflow can be further optimized to enhance proteome coverage. Increasing the number of replicates during SEC fractionation would improve total protein recovery across fractions, leading to more comprehensive downstream CZE and LC analyses and, consequently, greater overall proteoform identifications.^30,62^ Additionally, optimizing MS parameters, such as adjusting fragmentation energy, could improve the identification of high-mass proteoforms. Future studies examining truncation patterns could also help provide additional insight into the origin and functional significance of the detected proteoforms. Implementing a native CZE-MS/MS approach could further extend coverage to extremely large *E. coli* proteoforms, providing a more complete representation of the *E. coli* proteoforms.^63,64^ Additional studies focusing on the membrane proteins will also be necessary to further advance the proteome coverage. Overall, the draft map of *E. coli* proteoforms established here lays the foundation for future large-scale multidimensional TDP analyses of more complex biological samples (i.e., human cells, tissues, and biofluids).

## Supplementary Material





## Data Availability

The MS raw files have been deposited to the ProteomeXchange Consortium via the PRIDE partner repository with the data set identifier PXD078301.^71^

## Notes

Physicochemical properties of identified proteoforms across extraction buffers (DPBS, urea, and SDS) and separation methods (CZE and LC); distributions of identified proteoform families and proteoforms across SEC fractions obtained from different lysis buffers; higher-mass proteoform identifications with ProSight Native; GO enrichment analysis of the BUP and TDP data sets (PDF)
Lists of identified proteoforms and proteins from TDP and BUP analyses of *E. coli* cells; proteoform confidence classification (XLSX)
