# Supplementary material for: A Draft Map of E. coli Proteoforms
Source: Anal Chem. 2026 Aug 25;98(35):25961–71. doi: 10.1021/acs.analchem.6c03571 (PMC13564038; doi:10.1021/acs.analchem.6c03571)
Supplement: Supplementary file 1 [file ac6c03571_si_001.pdf]

A draft map of *E. coli* proteoforms

Jorge A. Colón-Rosado, Fei Fang, and Liangliang Sun*

Department of Chemistry, Michigan State University, 578 S Shaw Lane, East Lansing, Michigan 48824 United States

*Corresponding Author. Email: lsun@chemistry.msu.edu

Phone: 517-353-0498

Table of Contents

Supplementary Figure 1. Physicochemical properties of identified proteoforms across extraction buffers and separation methods S3

Supplementary Figure 2. Distributions of identified proteoform families and proteoforms across SEC fractions obtained from different lysis buffers S4

Supplementary Figure 3. Mass spectra and fragmentation pattern of higher mass (>30 kDa) proteoforms...S5

Supplementary Figure 4. Heatmaps of GO enrichment analysis of BUP and TDP datasets S6

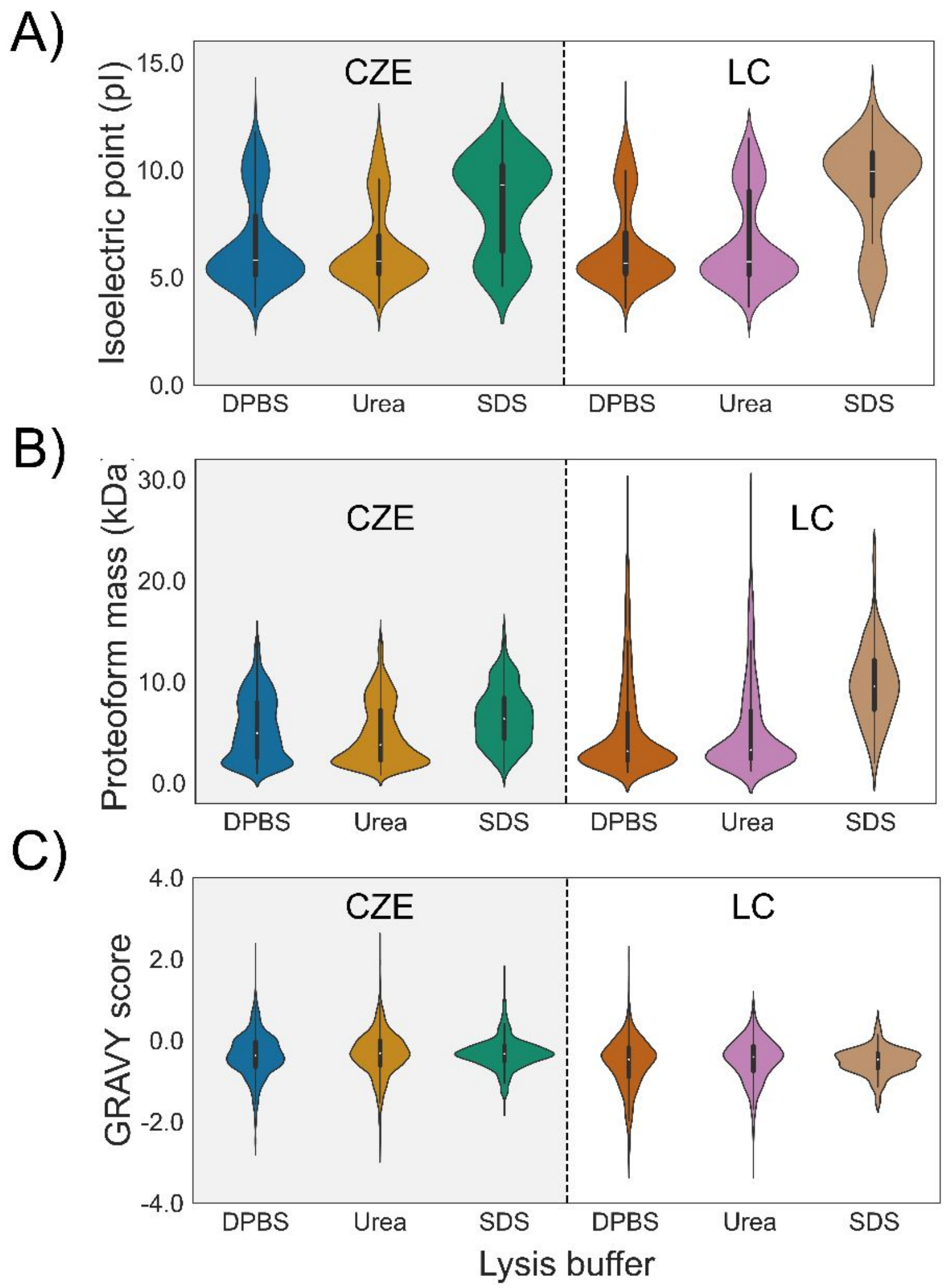

Figure S1. Physicochemical properties of identified proteoforms across extraction buffers (DPBS, urea, and SDS) and separation methods (CZE and LC). Violin plots showing A) pI, B) mass, and C) GRAVY score distributions of identified proteoforms. The GRAVY score tells the hydrophobicity of each proteoform. Positive GRAVY values indicate hydrophobic, and negative GRAVY values suggest hydrophilic.

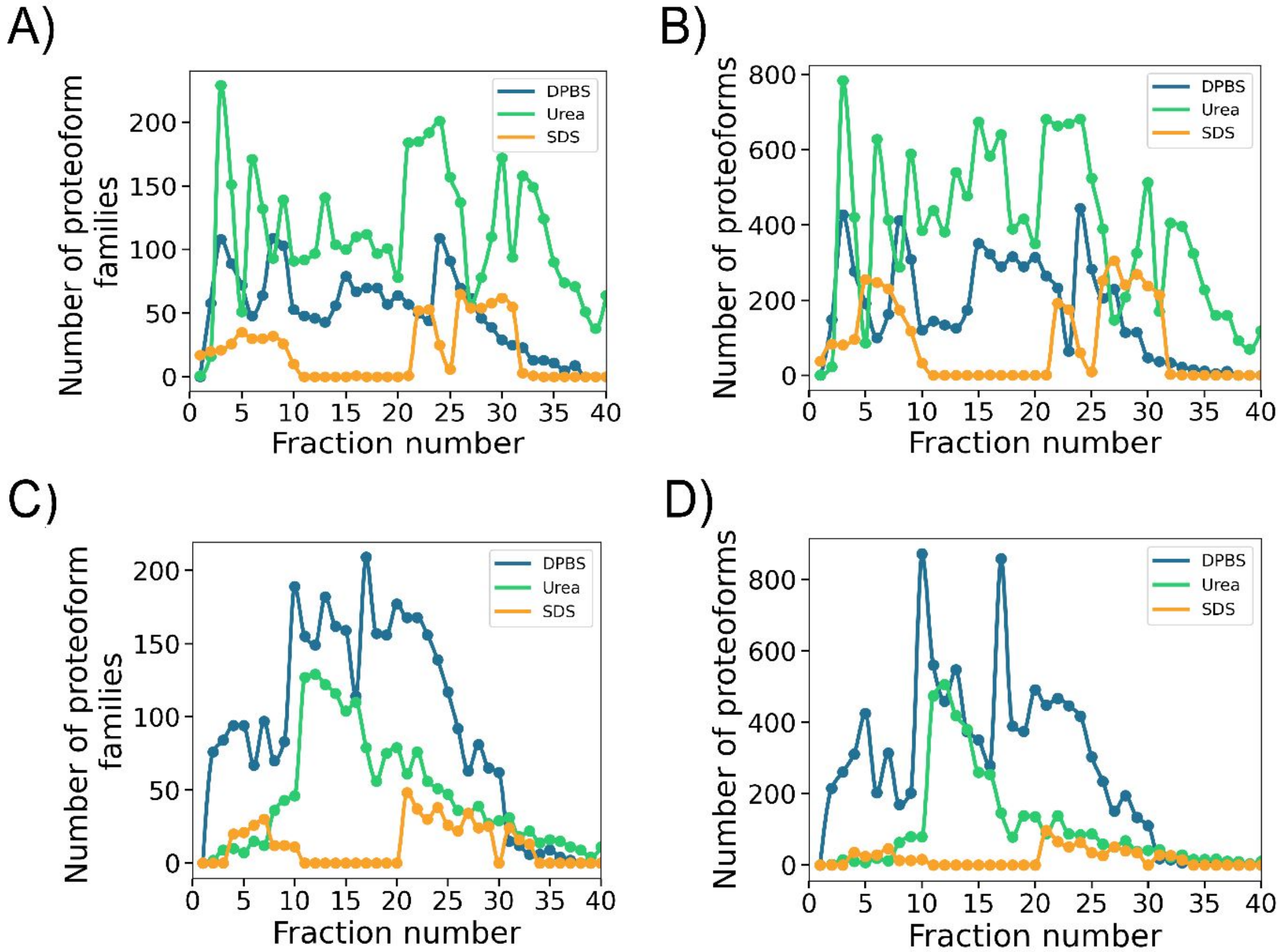

Figure S2. Distributions of identified proteoform families and proteoforms across SEC fractions obtained from different lysis buffers. The number of proteoform families and proteoforms identified in each fraction is shown for DPBS (blue), urea (green), and SDS (orange) extracts. A) and B) show proteoform families and proteoform identifications from CZE-MS/MS, whereas C) and D) show the data from RPLC-MS/MS.

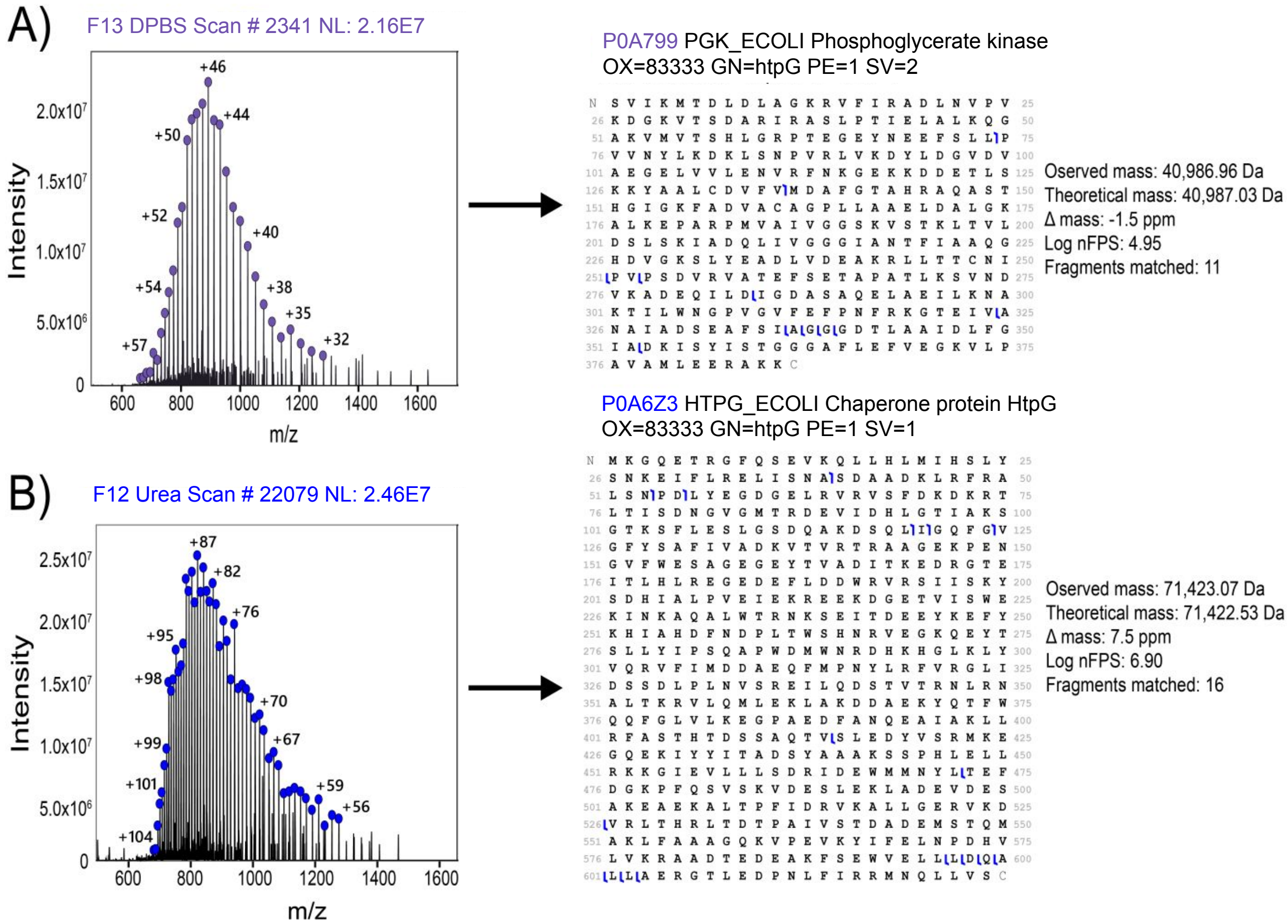

Figure S3. Higher mass proteoform identifications with Prosight Native. kDecon in Prosight Navite was used to deconvolve the MS1 spectra of two proteoforms, resulting in proteoforms masses of A) 40,986.96 and B) 71,422.53 Da. Fragmentation search within Prosight Native identified these proteoforms as A) phosphoglycerate kinase (P0A799) and B) chaperone protein HtpG (P0A6Z3).

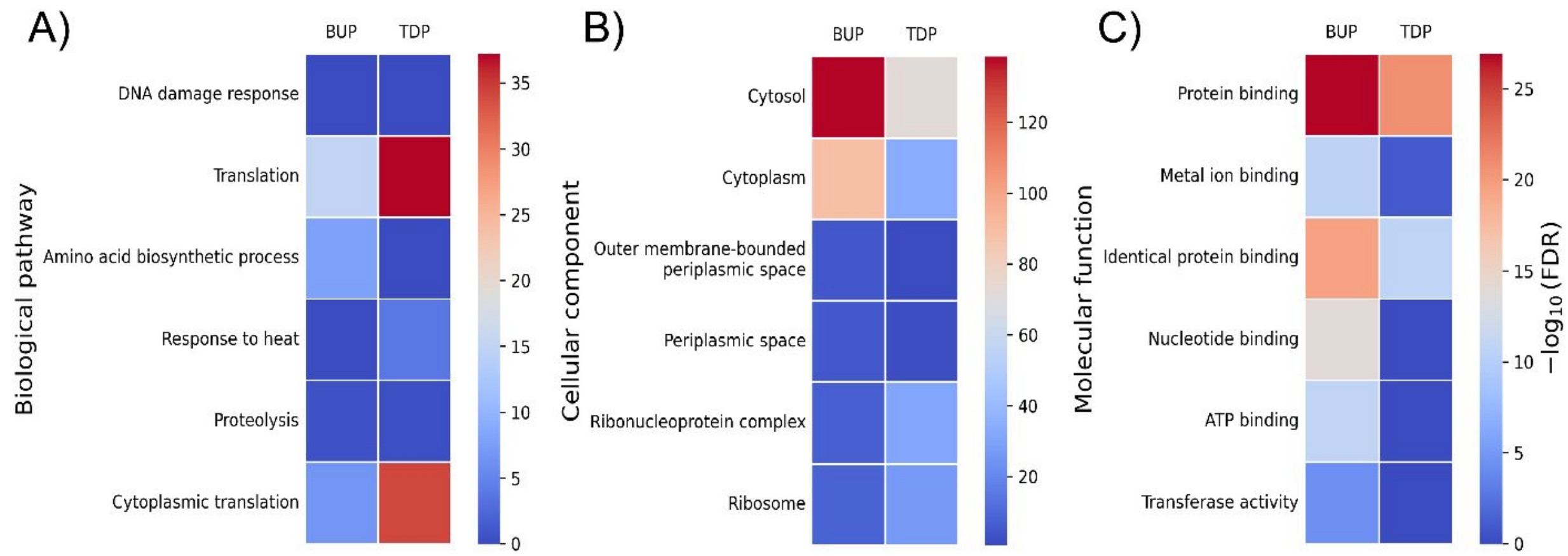

Figure S4. GO enrichment analysis of the BUP and TDP datasets. Heatmaps show enriched GO terms for A) biological process, B) cellular component and C) molecular function categories. Enrichment significance is represented as -$\log_{10}$(FDR).
